# The effect of multistrain probiotics on functional constipation in the elderly: a randomized controlled trial

**DOI:** 10.1038/s41430-022-01189-0

**Published:** 2022-08-04

**Authors:** Katarina Fehir Šola, Sanda Vladimir-Knežević, Pero Hrabač, Iva Mucalo, Luciano Saso, Donatella Verbanac

**Affiliations:** 1 Community Pharmacy Bjelovar, Bjelovar, Croatia; 2 University of Zagreb Faculty of Pharmacy and Biochemistry, Zagreb, Croatia; 3 Andrija Stampar School of Public Health, University of Zagreb School of Medicine, Zagreb, Croatia; 4 Department of Physiology and Pharmacology “Vittorio Erspamer”, Sapienza University of Rome, Rome, Italy

**Keywords:** Nutritional supplements, Geriatrics, Randomized controlled trials

## Abstract

**Background and objectives:**

Constipation is one of the most common gastrointestinal conditions, particularly among older individuals. This study aimed to evaluate the efficacy and safety of selected multistrain probiotics on functional constipation and laboratory blood parameters in the elderly living in a nursing home.

**Subjects and methods:**

Sixty participants (42 females and 18 males) aged 77.9 ± 8.84 years with functional constipation, who met the eligibility criteria, completed the study. In a double-blind, placebo-controlled, parallel design, each participant was randomized to receive either the selected probiotic mixture (*N* = 28) or placebo (*N* = 32) for 12 weeks as an adjunct to their usual diet and medications. The liquid probiotic formulation containing *Bifidobacterium animalis* subsp. *lactis* BLC1, *Lactobacillus acidophilus* LA3 and *Lactobacillus casei* BGP93 was tested for the first time.

**Results:**

Supplementation of selected probiotics resulted in a slight but nonsignificant increase in cumulative stool frequency compared with placebo. However, after the 71st day of the treatment, the cumulative number of stools was significantly higher in the probiotic group (*P* < 0.05) when the influence of laxative was excluded. The trend towards an increase in the difference between the two groups, which began 1 week after the probiotic intervention, pointed out to their prolonged effect. There were no significant dependent or independent effects of treatment and time on most of the 27 laboratory blood parameters tested.

**Conclusions:**

Multistrain probiotic supplementation was found to be efficacious, safe and well tolerated in the elderly with functional constipation.

## Introduction

Constipation is one of the most common chronic conditions among older individuals. Its prevalence rises with age and is more common in women. In some studies of self-reported constipation, 26% of women and 16% of men 65 years or older considered themselves to be constipated, whereas in the 84-year-old subgroup of patients the proportion of affected ones increased to 34% in women and 26% in men [1]. Constipation is even more frequent among nursing home residents. Several descriptive studies showed that about half of the residents suffer from chronic constipation and 56–75% of them take laxatives regularly [2]. The number of chronic diseases and the high rate of polypharmacy are significantly related to constipation. Besides, the frailty in the elderly that is associated with immobility, inadequate food intake and dehydration also contributes to the onset of constipation [3]. The chronic constipation has a detrimental influence on health-related quality of life with a significant decline in both mental and physical conditions. It also represents an economic burden to patients and to national health services [4].

The pathophysiology of functional constipation is multifactorial and includes diet, colonic motility and absorption, anorectal motor and sensory function and behavioural and psychological factors [5]. Despite an increasing number of evidence-based studies demonstrating the efficacy of various therapies, nearly half of patients are still dissatisfied with their symptom improvement and concerned about safety, adverse effects, inconvenience and taste [6, 7]. In this context, an increase in the investigation of the effectiveness of probiotics in the treatment of constipation has been noted over the past decade. The most recent meta-analysis of 15 randomized controlled trials demonstrated that probiotics, such as *Bifidobacterium*, *Lactobacillus* and *Streptococcus* strains, alleviate functional constipation in adults by increasing stool frequency, intestinal transit time and stool consistency. Multispecies probiotics were found to have a more significant beneficial effect on constipation symptoms than single-species probiotics [8]. Thus, modifying the gut luminal environment with specific probiotic strains may affect motility and secretion in the gut and provide a benefit for patients with functional constipation [9]. In addition, recent evidence suggests that the gut microbiota plays a key role in chronic, low-grade inflammation as one of the most consistent biological features of both chronological aging and various age-related diseases. Composition profile differences of intestinal microflora have been found in studies comparing healthy older individuals with hospitalized or institutionalized elderly patients [10]. The clinical efficacy of probiotics has been found to be dependent on the microbial strain. Their mixtures appear to have superior efficiency to single-strain probiotic since the effect of multispecies probiotics may be complementary or synergistic [11–13].

Considering all the above, the present randomized controlled trial aimed to evaluate the efficacy and safety of multistrain probiotics on functional constipation and laboratory blood parameters in the elderly living in a nursing home. The hypothesis of the study was that probiotics have a beneficial effect on stool frequency in the elderly with chronic constipation. In the present study, we used for the first time the liquid probiotic formulation containing *Bifidobacterium animalis* subsp. *lactis* BLC1, *Lactobacillus acidophilus* LA3 and *Lactobacillus casei* BGP93.

## Subject and methods

### Study participants

A total of 67 elderly nursing home residents aged 65 years or more, with functional constipation defined according to the Rome IV criteria and able to understand the procedure, were eligible for inclusion in the study. Subjects with diagnosis or history of obstructive ileus, suspected or confirmed diagnosis of irritable colon syndrome, ulcerative colitis, Crohn’s disease or malignant digestive tract disease, diarrhoea of any cause within the last month, acute infectious diseases not treated with antibiotics within the past month and opioid analgesics in pharmacotherapy were excluded from the study. Residence home users suffering from functional constipation and eligible for inclusion in the study were referred to the protocol details. Their voluntary participation was confirmed by signing the Informed consent after the objectives and the potential benefits and risks of the research were explained to them.

### Supplemented formulation composition

The probiotic preparation contained *Lactobacillus acidophilus* LA3 (1 × 10^9^ CFU/g), *Bifidobacterium animalis* subsp. *lactis* BLC1 (1.5 x 10^9^ CFU/g) and *Lactobacillus casei* BGP93 (2 × 10^9^ CFU/g) in the form of a liquid oral formulation to facilitate oral administration of nine drops with food. It was manufactured by PharmaS Ltd. Zagreb, Croatia (available on Croatian market under the name PROBalans SENIOR drops) and has never been studied formally in any patient cohort. Placebo treatment contained medium-chain triglyceride oil, fractionated oil obtained from coconut or palm oil with an effective content of triglycerides of caprylic (C8) and capric acid (C10) + silicon dioxide, and was of the same appearance, pharmaceutical form, and route of administration as the active treatment.

A daily dose of nine drops contains not less than 1 × 10^9^ CFU of probiotics at manufacture date. According to studies listed in the safety and efficacy report, this dosage has shown positive effects on the human gut. To ensure the stability of the product throughout its shelf life, the starting dose at the time of manufacture is closer to 6.25 × 10^9^ CFU/9 drops (based on the amount of each probiotic used and its declared strength in the material used in the manufacture). As the product is a powder suspended in an oil base, to ensure the recommended daily dosage of not less than 1 × 10^9^ CFU, nine drops have been chosen as the daily dose based on the density of the prepared suspension and the bottle assembly used at PharmaS Ltd in the manufacturing of this product.

### Study design and protocol

A single-centre, randomized, double-blind, placebo-controlled trial was conducted from December 2018 to February 2019 at Saint Camillus de Lellis nursing home, Vrbovec, Croatia. The first study phase was a recruitment period during which subjects were monitored primarily to ascertain the frequency of bowel discharges and general health, with no additional therapy being administered. After the first 4 weeks, they were re-evaluated for inclusion and exclusion criteria and the eligible patients were provided details about the study procedure. Patients who fulfilled the inclusion criteria proceeded to the second phase and were randomized to either the placebo (*N* = 32) or probiotic (*N* = 28) arm of the study. Randomization to intervention was conducted using a computer-generated algorithm. Participants, investigators, and statisticians were blinded to the identity of the probiotic and placebo by coding and by the indistinct nature of the liquid formulation.

Participants were instructed to take nine drops of a liquid oral formulation once a day (prior to the main meal) for the next 12 weeks, in addition to their current therapy. The study duration of 12 weeks was chosen primarily based on our previous experience with the test product, which showed that initial effects occurred after a few weeks of treatment, while full effects were expected after about 10 weeks. Stool frequency was monitored and recorded daily in each individual respondent including an additional week to assess the prolonged effect of the probiotic intervention. The primary response variable was the cumulative number of stools for each subject over the study period. All other variables were considered secondary. Participants underwent two sessions of venous blood sampling at the baseline and after 12 weeks of the intervention. Collected blood samples were analyzed for 27 laboratory blood parameters listed in Table 2: high-sensitivity C-reactive protein (hsCRP), glucose (Glu), vitamin B12 (B12), folic acid (Fol), triglyceride (TG), total cholesterol (TC), high-density lipoprotein (HDL), low-density lipoprotein (LDL), aspartate aminotransferase (AST), alanine aminotransferase (ALT), *gamma*-glutamyl transferase (GGT), alkaline phosphatase (ALP); and in Supplementary Table S3: white blood cells (WBC), red blood cells (RBC), haemoglobin (hbg), haematocrit (HCT), mean corpuscular volume (MCV), mean corpuscular haemoglobin (MCH), mean corpuscular haemoglobin concentration (MCHC), platelet (PLT), red cell distribution width (RDW), mean platelet volume (MPV), percentage of neutrophils (Neuts), percentage of lymphocytes (Lymphs), percentage of monocytes (Monos), percentage of eosinophils (Eos) and percentage of basophils (Baso). The rationale for monitoring such a large number of blood parameters was to evaluate the safety, i.e., we wanted to confirm that the test product would not cause major disturbances in blood parameters.

### Data collection

The research team, composed of three academic members, a general practitioner, a nurse and a community pharmacist-researcher, was responsible for conducting the work and checking the compliance against the inclusion criteria for each subject included in the study. Resident data were retrieved by reviewing their admission documents, medical records, and interviewing residents and the home medical staff. Standard demographic and anthropometric characteristics, current and past medical history, regular prescription and over-the-counter medicines, history of drug allergies and adverse drug reactions, serum biochemistry parameters, renal function and blood pressure were collected for each resident by a community pharmacist-researcher. Participants’ lifestyle habits (eating habits, physical activity, and fluid intake) and laxative bisacodyl was kept during the study. The nursing home nutritionist decided on the amount and type of food consumed. Individual differences were present due to participants’ personal preferences and the various concomitant diseases. However, the two study groups were fully comparable in terms of diet. The daily stool numbers of each individual respondent in the probiotic and placebo groups were recorded the next morning by the nursing staff and community pharmacist-researcher. Venous blood samples were taken after 10–12 h overnight fasting in the ambulance of the nursing home and analyzed at the Department of Laboratory Diagnostics, Clinical Hospital Dubrava, Zagreb.

### Statistical analysis

Sample Size Software (2017, NCSS, LLC, Kaysville, Utah, USA, ncss.com/software/pass.) was used for the purposes of sample size calculation analysis. Initial calculation was based on a pilot study performed in the same nursing home where the trial was performed. The pilot study included two groups of 8 residents who received either placebo or verum preparation, with the final aim of comparing cumulative number of stools after the 90-day trial period. Using the parameters of alpha=0.05 and power (1 – beta) of 0.90, the final sample size needed was estimated to be 21 persons per group.

The Statistica software package (TIBCO Software Inc., 2018) was used to analyze the data obtained from the study. Due to the large number of analyses performed, a correction for multiplicity was applied, with a level of statistical significance set to 0.01. For continuous variables, normality of distribution was checked by the Kolmogorov–Smirnov test, and appropriate parametric or non-parametric tests were used accordingly. The demographic findings and participant characteristics were compared using a t-test for independent samples. Results are expressed as number of participants (*N*), median (IQR) and significant at *P* < 0.05. The cumulative number of stools between two study groups was tested for each day using the Mann–Whitney U test. *P* values in Supplementary Tables S1 and S2 show the results of these tests.

## Results

### Baseline characteristics of the participants

Initially, 67 residents were assessed for their eligibility and enrolled in the study. Over the study period, seven participants passed away and were excluded from further analysis. None of the deaths could be attributed to the supplementation provided throughout the study. Sixty participants (42 females and 18 males) aged 78 (65–98) years, with functional constipation, who met the eligibility criteria, completed the study. They followed the study protocol without difficulty and reported no side effects following the consumption of either probiotic or placebo. Analysis of baseline parameters revealed that the two groups were similar in all demographic and anthropometric parameters (Table 1).

**Table 1:** Characteristics of the participants.

Variable	Placebo (*N* = 32)	Probiotic (*N* = 28)	*P* value
Gender [f, *N*/total]	21/32	21/28	0.429^a^
Age, years [median (IQR)]	79 (65–98)	76 (66–82)	0.198^b^
BMI, kg/m^2^ [median (IQR)]	26.6 (15.2–35.0)	28.3 (23.5–47.8)	0.304^b^
Formal education [*N*, ratio]			
None	5 (0.156)	6 (0.214)	
Elementary school	17 (0.531)	8 (0.286)	0.172^c^
High school	8 (0.25)	8 (0.286)	
University	2 (0.063)	6 (0.214)	
Marital status [*N*, ratio]			
Unmarried	6 (0.188)	7 (0.25)	
Married	3 (0.094)	4 (0.143)	0.310³
Divorced	2 (0.063)	5 (0.179)	
Widow/er	21 (0.656)	12 (0.429)	

^a^Fisher test.

^b^Mann–Whitney *U* test.

^c^Fisher–Freeman–Halton test.

### The effect of probiotics on functional constipation

**Fig. 1:**
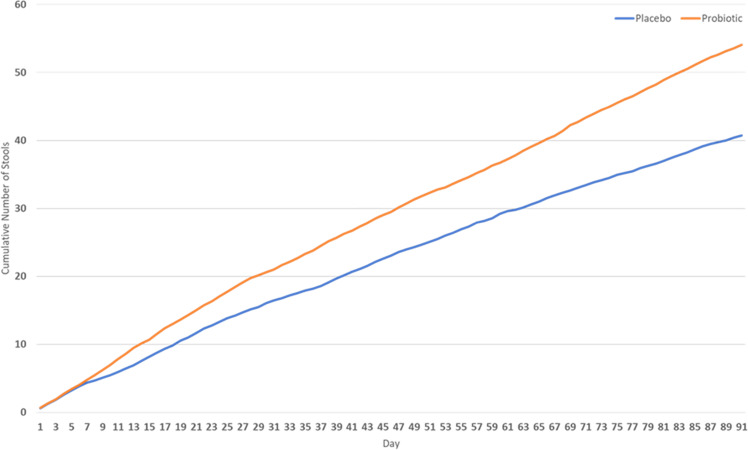
Basic comparison of cumulative numbers of stools between probiotic and placebo groups. After the first week, the cumulative number of stools in the probiotic group (blue line) is consistently higher compared to placebo (orange line). Although there is no statistically significant difference (*P* > 0.05), a trend of increasing difference between groups is visible.

**Fig. 2:**
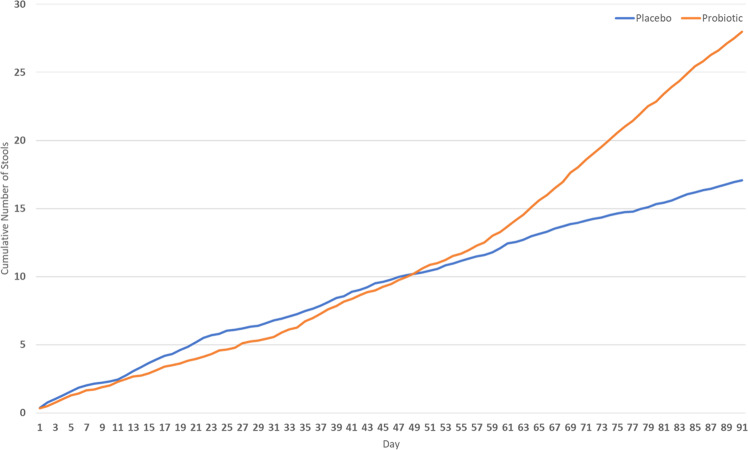
Comparative cumulative numbers of stools in probiotic and placebo groups without the influence of laxatives used. After the 75th day of the treatment, the difference between probiotic (blue line) and placebo (orange line) groups is statistically significant (*P* < 0.05).

The cumulative numbers of stools were comparable between the probiotic (mean consumption of 48; 31–70, cumulative number of stools 54) and placebo (mean consumption of 38; 29–53, cumulative number of stools 41) groups during the 91 days of the study (Fig. 1). The number of stools in the probiotic group was consistently higher compared to placebo after the first week, but there was no significant difference between the two groups. However, there was a trend towards an increasing difference between the groups. *P* values ranged from 0.994 (first week) to 0.090 (last week). The subjects in both groups took a similar number of laxatives with a mean consumption of 16 (9–28) in the placebo group (number of laxatives 33) and 18 (9–33) in the probiotic group (number of laxatives 29) (*P* = 0.665). Bisacodyl was commonly used to treat constipation, and other laxatives were used very rarely. After excluding the data for days when participants used laxatives, the cumulative numbers of stools in the groups were compared again. From the results shown in Fig. 2, similar cumulative values of stool numbers in both groups can be observed during the first 6 weeks. The difference between the groups began to increase in favour of probiotics and reached statistical significance after the 71st day of the treatment (*P* < 0.05). Complete data on *P* values are shown in supplementary materials (Supplementary Tables S1 and S2).

### The effect of probiotics on laboratory blood parameters

A total of 27 laboratory blood parameters were processed. Within- and between-treatment differences were assessed for probiotic and placebo groups in all blood parameters. Table 2 provides the values of 12 selected parameters, while the others are shown in Supplementary Table S3. Laboratory parameter values did not differ significantly between both interventions at baseline. In most cases, there was no significant dependent and independent effect of treatment and time on blood parameter changes. A significant within treatment change during probiotic use was found in both glucose (*P* = 0.018) and B12 (*P* = 0.019), as well as in cholesterol (*P* = 0.032) and LDL (*P* = 0.043) during placebo intervention (Supplementary Table S3).

**Table 2:** Selected laboratory blood parameters in placebo and probiotic groups.

Parameters	Placebo (*N* = 32)		Probiotic (*N* = 28)	
	Week 0	Week 12	Week 0	Week 12
hsCRP (mg/L)	3.6 (2.35–8.35)	3.7 (1.6–7.0)	1.9 (3.3–11.1)	5 (2.5–9.5)
Glu (mmol/L)	5.1 (4.3–5.65)	4.7 (4.4–5.4)	5.8 (4.4–5.4)	4.5 (3.9–6)
B12 (pmol/L)	191.5 (134–258)	198 (138–266)	202 (155–276)	245 (166–325)
Fol (nmol/L)	7.7 (4.9–11.05)	7.2 (5.6–14.2)	7.6 (6.1–10.4)	8.4 (6.7–10.3)
TG (mmol/L)	1.4 (1.1–1.7)	1.3 (1–1.6)	1.6 (1.1–2)	1.4 (1–2.2)
TC (mmol/L)	5.3 (4.25–6.2)	5.1 (4.2–6.2)	5.5 (4.9–6.2)	5.1 (4.4–6.2)
HDL (mmol/L)	1.1 (1–1.45)	1.2 (1–1.5)	1.1 (0.9–1.4)	1.1 (0.9–1.4)
LDL (mmol/L)	3.4 (2.3–4.3)	3.1 (2.4–4)	3.7 (2.7–4.3)	3.3 (2.7–4)
AST (U/L)	19.5 (17–24)	18 (16–24)	18 (16–23)	17 (15–22)
ALT (U/L)	14.5 (11–19.5)	12 (9–19)	13 (11–19)	14 (11–20)
GGT (U/L)	24 (19–32)	20 (17–27)	21 (19–32)	22 (17–29)
ALP (U/L)	80.5 (73–107.5)	82 (68–108)	81 (69–99)	86 (74–98)

Data expressed as median (IQR).

*hsCRP* high-sensitivity C-reactive protein, *Glu* glucose, *B12* vitamin B12, *Fol* folic acid, *TG* triglyceride, *TC* total cholesterol, *HDL* high-density lipoprotein, *LDL* low-density lipoprotein, *AST* aspartate aminotransferase, *ALT* alanine aminotransferase, *GGT* gama-glutamyl transferase, *ALP* alkaline phosphatase.

*P < 0.05.

### Safety and tolerability

There were no significant differences in haematological or biochemical safety parameters between placebo and probiotic groups over the study period (Table 2 and Supplementary Table S3). Mean values were within clinical reference ranges before and after the intervention. No adverse events were observed in either group. Tolerability was excellent, with 100% of samples tested in both groups throughout the study and no dropouts due to probiotic or placebo consumption.

## Discussion

To our knowledge, this is the first clinical trial to demonstrate the efficacy and safety of the liquid probiotic formulation containing *Bifidobacterium animalis* subsp. *lactis* BLC1, *Lactobacillus acidophilus* LA3 and *Lactobacillus casei* BGP93. This multispecies probiotic combination was shown to a trend to increase the stool frequency in elderly nursing home residents, although it did not result in statistical significance. Our primary outcome was to examine the effect of selected probiotic strains on the cumulative number of stools. Although there was no significant difference between the two groups, the cumulative number of stools in the probiotic group was consistently higher than placebo and *P* values tended to decrease gradually from the second week to the end of the intervention period. Moreover, when cumulative stool numbers were compared between the two groups with the exclusion of data when respondents used laxatives, a statistically significant difference was reached in the tenth week of the treatment. The trend of increasing the difference between the two groups, one week after the probiotic intervention indicated their prolonged effect. Our results suggested that a long-term intake of these probiotics is needed to achieve a significant effect on constipation symptoms and their effectiveness may be reduced by the concomitant use of laxative drugs. This is in line with recent findings that combination therapy of a probiotic and laxative did not demonstrate any additive effects in children with functional chronic constipation [14]. Although our major goal was to evaluate how a selected probiotic mixture affected functional constipation in a real-life experiment without changing lifestyle habits, such as regular diet and laxative intake, it is important to emphasize that constipation may be significantly influenced by dietary fibre consumption. In a recent meta-analysis, a high-fibre diet was linked to a lower prevalence of constipation [15]. Another meta-analysis found that short-chain β-fructan supplementation significantly increased the frequency of bowel movements [16–18].

Recent evidence supports the role of the gut microbiota in the pathogenesis and treatment of functional constipation. A decreased level of bifidobacteria and lactobacilli and an increased number of *Bacteroidetes* were noted in the constipated subject compared to healthy controls [15, 19]. However, previous clinical trials have given mixed results regarding the effect of probiotics on functional constipation, which could be in part due to the different strains and doses of probiotics administered, clinical heterogeneity of participants, as well as design and duration of the trials. Studies conducted with the BB-12 and GCL2505 strains of *Bifidobacterium animalis* subsp. *lactis* (syn. *Bifidobacterium lactis*) resulted in a clinically relevant benefit in increasing the amount of total bifidobacteria in the human intestine [14], as well as defecation frequency and abdominal discomfort in healthy subjects with low defecation frequency, respectively [20, 21]. The dose-response effect of *Bifidobacterium lactis* HN019 on whole gut transit time and functional gastrointestinal symptoms in adults was revealed [15], but not confirmed in another study [22]. The recent randomized clinical trial demonstrated that supplementation with *Bifidobacterium lactis* NCC2818 did not result in a significant reduction in the whole gut transit time, nor impact other constipation‐related outcomes and changes in stool microbiota in adults [23]. Consumption of a probiotic beverage containing *Lactobacillus casei* Shirota resulted in a significant improvement in self-reported severity of constipation and stool consistency in patients with chronic constipation [24]. A few formulations composed of different strains of bifidobacteria and lactobacilli have also been included in clinical trials also. Although there was no statistical significance, the multistrain probiotic blend consisting of *Lactobacillus acidophilus* DDS-1, *Bifidobacterium animalis* subsp. *lactis* UABla-12, *Bifidobacterium longum* UABl-14 and *Bifidobacterium bifidum* UABb-10 helped modulate bowel function earlier than the placebo, with a corresponding shift to a more fibrolytic microbiota [25, 26]. Furthermore, the product containing yogurt with polydextrose, *Bifidobacterium lactis* HN019 and *Lactobacillus acidophilus* NCFM significantly shortened colonic transit time compared with the control [24].

Our study initially hypothesized that the observed beneficial effects of probiotics in the elderly with chronic constipation were related to modulation of the age-related imbalance of the gut microbiota and improvement of the immune response, which was the secondary outcome. A common feature of ageing is a reduction in innate and acquired immune function followed by an increase in the concentrations of prototypical proinflammatory mediators in the bloodstream, such as acute phase proteins, cytokines, and adhesion molecules (‘inflammageing’) [27]. This phenomenon is a highly substantial risk factor for morbidity and mortality in the elderly. Many possible triggers for low-grade inflammation have been proposed, ranging from dysfunctional mitochondria and consequent oxidative stress to an imbalance in the gut microbiota. Therapeutic manipulation of intestinal bacteria by selectively altering the beneficial versus harmful microbial species could reverse the inflammatory responses and restore mucosal homoeostasis [7, 27, 28]. hsCRP is recognized as an exquisitely sensitive systemic marker of inflammation, infection, and tissue damage. Many studies have shown that elevated serum hsCRP levels are associated with ageing and ageing-related diseases [29, 30]. Our study also demonstrated that most respondents had elevated serum hsCRP levels (>3 mg/L) indicating the presence of chronic low-grade inflammation. Although hsCRP levels were decreased in the probiotic group compared with placebo, this was not statistically significant. These findings are not completely in line with the results of a recent meta-analysis which showed that probiotics significantly reduce serum concentrations of hsCRP and proinflammatory cytokines [31]. In contrast, some studies reported a nonsignificant effect of probiotic supplementation on serum hsCRP levels [32].

The prevalence of vitamin B12 deficiency increases with age, particularly in the frail and institutionalized subjects, but is often unrecognized because its clinical manifestations are subtle. It could be potentially serious, especially from neuropsychiatric and haematological perspectives [33, 34]. Probiotic bacteria, mostly belonging to the genera *Lactobacillus* and *Bifidobacterium*, provide numerous health benefits to the host, including vitamin B production [35]. Considering that, probiotic and placebo groups were compared and a significantly higher increase in B12 levels was found after 12 weeks of treatment with tested multistrain probiotics (*P* = 0.019). In contrast, there was no treatment effect on folate as the most common vitamin deficiency in later life, too (Table 2).

As known from previous investigations, consumption of certain probiotic strains could improve lipid metabolism in various diseases and conditions [36, 37]. Our probiotic intervention did not affect TC, LDL and HDL cholesterol levels, which is consistent with the results of some other studies [38, 39]. The recent meta-analysis demonstrated that probiotic interventions reduced TC and LDL levels in hypercholesterolaemic adults, but no significant effects of probiotics on triglyceride and HDL levels were found. Subgroup analyses indicated that the efficacy of probiotics varied by strain [40].

A positive impact of bifidobacteria and lactobacilli on metabolic control in patients with hyperglycaemia has been reported [41, 42]. One meta-analysis suggested that probiotic supplementation may be beneficial in lowering fasting blood glucose (FBG) in adults with high baseline levels (≥7 mmol/L) and that multispecies probiotics may have more impact on FBG than single species [43]. Accordingly, our research showed a mild, but significant reduction in FBG levels after probiotic supplementation (*P* = 0.019).

The probiotic mixture tested in this study was shown to be well tolerated and safe to consume during the 12-week treatment period according to haematological, biochemical, and adverse events profiles, which is in line with data previously reported by others [12, 25, 44].

Among the limitations of the study, the most important one is the limited number of subjects included, which is a consequence of a single-centre design. This design was chosen despite its shortcomings because we wanted to have as homogenous population as possible in terms of age, gender, habits, medical history and other relevant parameters. Differences in daily care protocols, diet and concomitant medication consumption were other arguments in favour of a single-centre design over the multicentre approach. Although this meant that the number of subjects included was limited, we believe that the trade-off between the number of subjects and the homogeneity of the population balances this decision. We were also guided by the fact that a comparison of the cumulative number of stools between the groups was possible because the size of the study group, the total amount of laxatives taken, and the duration of the study were comparable between the two study groups.

Our findings suggested that selected multistrain probiotics can be beneficial as an adjunctive therapeutic tool to treat functional constipation in elderly nursing home residents. Presented results are consistent with those of others that supported superior effects on constipation symptoms of multistrain probiotic supplements in comparison to single probiotics, which could be attributed to synergistic interactions between distinct strains with varied activities [8]. Supplementation with a new and original liquid formulation containing *Bifidobacterium animalis subsp. lactis* BLC1*, Lactobacillus acidophilus* LA3 and *Lactobacillus casei* BGP93 was proved to be effective, safe and well tolerated. The initial hypothesis of the trial was demonstrated by statistical analysis of the cumulative number of stools in two study groups. The primary analysis of interest was the comparison of the number of stools on days when subjects did not take laxatives. The *P* values listed in the supplementary materials (Supplementary Table S2) clearly confirm the statistical significance of the differences between the groups. However, further long-term, multicentre, randomized, controlled studies are required to elucidate the impact of selected probiotics on stool frequency and transit time as well as on the gut microbiota and low-grade inflammation in older adults with functional constipation.

## Supplementary information

Table S1

Table S2

Table S3

## Data Availability

Data can be found within the published article and its supplementary files. Requests for materials should be addressed to KFŠ.
