# Supplementary material for: The effect of multistrain probiotics on functional constipation in the elderly: a randomized controlled trial
Source: Eur J Clin Nutr. 2022 Aug 4;76(12):1675–81. doi: 10.1038/s41430-022-01189-0 (PMC9708599; doi:10.1038/s41430-022-01189-0)
Supplement: Supplementary file 1 — Table S1 [file 41430_2022_1189_MOESM1_ESM.docx]

| **Table S1: Results (p-values) of statistical testing of differences in cumulative number of stools between placebo and probiotic groups over the study period** | | | | | | | | |
| --- | --- | --- | --- | --- | --- | --- | --- | --- |
| **D1-10** | **D11-20** | **D21-30** | **D31-40** | **D41-50** | **D51-60** | **D61-70** | **D71-80** | **D81-91** |
| 0,994 | 0,103 | 0,175 | 0,173 | 0,136 | 0,126 | 0,212 | 0,129 | 0,090 |
| 0,959 | 0,094 | 0,164 | 0,152 | 0,119 | 0,124 | 0,182 | 0,131 | 0,092 |
| 0,913 | 0,107 | 0,155 | 0,138 | 0,114 | 0,164 | 0,175 | 0,126 | 0,097 |
| 0,943 | 0,142 | 0,158 | 0,132 | 0,138 | 0,167 | 0,175 | 0,127 | 0,094 |
| 0,994 | 0,175 | 0,186 | 0,132 | 0,148 | 0,175 | 0,182 | 0,126 | 0,100 |
| 0,932 | 0,205 | 0,140 | 0,115 | 0,156 | 0,199 | 0,186 | 0,121 | 0,096 |
| 0,696 | 0,181 | 0,147 | 0,107 | 0,138 | 0,189 | 0,191 | 0,116 | 0,097 |
| 0,442 | 0,141 | 0,136 | 0,097 | 0,148 | 0,160 | 0,169 | 0,115 | 0,099 |
| 0,248 | 0,172 | 0,146 | 0,120 | 0,129 | 0,162 | 0,142 | 0,109 | 0,103 |
| 0,156 | 0,136 | 0,157 | 0,125 | 0,120 | 0,204 | 0,126 | 0,100 | 0,105 |
|  |  |  |  |  |  |  |  | 0,097 |

*D = day; Level of significance is marked by colour gradient with 0,999 being dark green*
