# Supplementary material for: The effect of multistrain probiotics on functional constipation in the elderly: a randomized controlled trial
Source: Eur J Clin Nutr. 2022 Aug 4;76(12):1675–81. doi: 10.1038/s41430-022-01189-0 (PMC9708599; doi:10.1038/s41430-022-01189-0)
Supplement: Supplementary file 2 — Table S2 [file 41430_2022_1189_MOESM2_ESM.docx]

**Table S2: Results (p-values) of statistical testing of differences in cumulative number of stools between placebo and probiotic groups on day without laxatives**

| **D1-10** | **D11-20** | **D21-30** | **D31-40** | **D41-50** | **D51-60** | **D61-70** | **D71-80** | **D81-91** |
| --- | --- | --- | --- | --- | --- | --- | --- | --- |
| 0,563 | 0,542 | 0,111 | 0,172 | 0,676 | 0,766 | 0,646 | 0,033 | 0,001 |
| 0,145 | 0,419 | 0,097 | 0,252 | 0,750 | 0,837 | 0,449 | 0,028 | 0,001 |
| 0,215 | 0,340 | 0,113 | 0,285 | 0,794 | 0,955 | 0,351 | 0,020 | 0,001 |
| 0,300 | 0,205 | 0,153 | 0,312 | 0,666 | 0,893 | 0,319 | 0,012 | 0,001 |
| 0,366 | 0,131 | 0,110 | 0,412 | 0,740 | 0,972 | 0,264 | 0,007 | 0,000 |
| 0,231 | 0,134 | 0,149 | 0,462 | 0,729 | 0,921 | 0,214 | 0,004 | 0,001 |
| 0,229 | 0,179 | 0,208 | 0,506 | 0,876 | 0,860 | 0,191 | 0,004 | 0,000 |
| 0,175 | 0,184 | 0,193 | 0,506 | 0,899 | 0,740 | 0,164 | 0,003 | 0,000 |
| 0,310 | 0,140 | 0,181 | 0,571 | 0,972 | 0,677 | 0,094 | 0,002 | 0,000 |
| 0,369 | 0,152 | 0,168 | 0,692 | 0,848 | 0,734 | 0,068 | 0,003 | 0,000 |
|  |  |  |  |  |  |  |  | 0,000 |

*D = day; Level of significance is marked by colour gradient with 0,999 being dark green and <0,001 being intensive red.*
