# Supplementary material for: The effect of multistrain probiotics on functional constipation in the elderly: a randomized controlled trial
Source: Eur J Clin Nutr. 2022 Aug 4;76(12):1675–81. doi: 10.1038/s41430-022-01189-0 (PMC9708599; doi:10.1038/s41430-022-01189-0)
Supplement: Supplementary file 3 — Table S3 [file 41430_2022_1189_MOESM3_ESM.docx]

**Table S3: Selected laboratory blood parameters in placebo and probiotic groups**

| **Parameters** | **Week 0** | | | **Week 12** | | |
| --- | --- | --- | --- | --- | --- | --- |
|  | **Placebo** | **Probiotic** | **P** | **Placebo** | **Probiotic** | **P** |
|  |  |  |  |  |  |  |
| WBC(x10^9^/L) | 8.01±2.37 | 8.00±2.5 | 0.783 | 7.46±2.14 | 7.45±1.93 | 0,962 |
| RBC (x10^12^/L) | 4.35±0.59 | 4.41±0.58 | 0.608 | 4.3±0.5 | 4.41±0.73 | 0.355 |
| Hbg(g/L) | 132.34±17.31 | 129.55±15.67 | 0.319 | 129.55±19.14 | 129.52±18.87 | 0.956 |
| HCT(L/L) | 0.4±0.05 | 0.4±0.05 | 0.515 | 0.4±0.06 | 0.39±0.06 | 0.743 |
| MCV(fL) | 92.26±6.82 | 90.51±6.45 | 0.199 | 92.29±8.37 | 90.87±6.57 | 0.187 |
| MCH(pg) | 30.31±2.30 | 29.66±2.45 | 0.322 | 30.03±3.29 | 31.97±11.9 | 0.463 |
| MCHC(g/L) | 325.63±17.40 | 328.76±8.25 | 0.873 | 325.97±18.8 | 317.07±57.21 | 0.882 |
| RDW(%) | 14.37±1.03 | 14.66±1.18 | 0.465 | 18.41±21.18 | 15.24±1.64 | 0,177 |
| PLT(x10^9^/L) | 250.63±61.26 | 274.31±71.31 | 0.172 | 237.81±60.46 | 265.37±82.46 | 0.268 |
| MPV(fL) | 8.99±1.01 | 9.41±4.07 | 0.308 | 8.77±1.12 | 8.75±1.06 | 0.803 |
| Neuts(%) | 56.91±7.01 | 54.9±10.3 | 0.359 | 60.38±9.88 | 56.37±8.88 | 0.254 |
| Lymphs(%) | 30.65±6.77 | 31.35±11.18 | 0.998 | 29.38±15.57 | 30.07±9.29 | 0.391 |
| Monos(%) | 7.30±1.73 | 7.71±1.75 | 0.374 | 7.08±2.14 | 7.74±2.03 | 0.236 |
| Eos(%) | 3.54±2.11 | 3.67±2.76 | 0.649 | 3.04±2.05 | 3.37±2.86 | 0.318 |
| Baso(%) | 0.67±0.38 | 0.68±0.35 | 0.655 | 0.85±0.68 | 0.77±0.52 | 0.803 |

Parameters: white blod cells (WBC), red blod cells (RBC), heamoglobin (hbg), hematrocrit (HCT), mean corpuscular volume (MCV), mean corpuscular haemoglobin (MCH), mean corpuscular haemoglobin concentration (MCHC), platelet (PLT), red cell distribution width (RDW), mean platelet volume (MPV), percentage of neutrophils (Neuts), percentage of lymphocytes (lymphs), percentage of monocytes (monos), percentage of eosinophils (eos) and percentage of basophils (baso).
